# Editorial: *In vivo* and *in situ* imaging for characterization of colorectal cancer

**DOI:** 10.3389/fgstr.2024.1363016

**Published:** 2024-01-25

**Authors:** Sean Benson, Frauke Alves

**Affiliations:** ^1^ Department of Radiology, The Netherlands Cancer Institute (NKI), Amsterdam, Netherlands; ^2^ Department of Cardiology, Amsterdam University Medical Center, Amsterdam, Netherlands; ^3^ Department of Diagnostic and Interventional Radiology, University Medical Center Göttingen, Göttingen, Germany; ^4^ Translational Molecular Imaging, Max Planck Institute for Multidisciplinary Sciences, Goettingen, Germany

**Keywords:** imaging, histopathology, MRI, computed tomography, artificial intelligence, radiomics, deep learning

## 1 Introduction

Our Research Topic focuses on the imaging of colorectal cancer at all scales, with the goal of being able to identify imaging biomarkers predictive of disease progression and therapy resistance. The Research Topic is intentionally very broad in terms of imaging modalities, as the heterogeneity of tumors and the associated microenvironment requires high-resolution 2D and 3D imaging of tissue biopsies together with clinical tomographic imaging to comprehensively characterize this complex cancer type in more detail. Our topic shows the complementary insights offered by microscopy and non-invasive radiological modalities such as computed tomography (CT) and magnetic resonance imaging (MRI). In addition, our topic also provides a glimpse into modern analysis techniques such as artificial intelligence (AI) and deep learning in order to identify novel imaging features for improved classifications of cancer subtypes.

## 2 Aims and objectives

The main aim of this Research Topic is to showcase the real actionable information that can be derived from multimodal imaging strategies in colorectal tumors, whether for better staging, metastatic risk, or assessment of nodal involvement. Of particular interest to the topic are novel 3D imaging strategies such as X-ray based virtual histology and other modalities that minimally disturb the microenvironment of the tumor and visualize the entire biopsy in 3D. It is also an objective of the Research Topic to assess non-invasive imaging methods that are capable of producing features that can enable predictions for future prospective clinical studies, in which AI is taking an increasingly predominant role.

## 3 Contributing articles: a mosaic of modalities

Conventional histological images provide a wealth of information about the biopsied tumor. However, proper manual study and annotation is a laborious task for such high resolution images. A novel deep learning method involving generative adversarial networks is shown by Jiang et al. to be effective in improving tissue type classification, using synthetic data to augment the training set. In contrast to 2D histology images,Svetlove et al. presents X-ray phase contrast 3D virtual histology images of paraffin-embedded colon tumor specimens with a voxel resolution of 2*μ*m using the SYRMEP beamline of the Italian synchrotron Elettra. X-ray-based microcomputed tomography represents a non-destructive isotropic 3D imaging technique, which is especially important to assess the tumor microenvironment with different cell types, extracellular matrix, fat and vessels with the entire biopsy intact. While we have seen from the work of Jiang et al. that 2D histopathology provides impressive classification possibilities, clinically important features such as tumor budding, the invasion front, and vascular infiltration are morphological characteristics typically not easy to assess. Experienced pathologists can obtain this information using multiple 2D histologic slices. However, a true 3D representation would undoubtedly be preferred.

Tomographic *in vivo* imaging methods such as CT and MRI do not have the same resolution as histopathology and typically achieve a spatial resolution of around a millimeter for 3T MRI scanners. However, the generation of such images is far less invasive and does not require a biopsy. Indeed in the case of MRI there is no exposure to ionizing radiation, which provides an added benefit. Using features derived from MRI in association with artificial intelligence, Qu et al. shows that it is possible to predict tumor budding, which is a prognostic factor according to the official American Joint Committee on Cancer rectal cancer guidelines. Given that tumor budding is a post-operative pathological characteristic, the ability to predict it with non-invasive baseline imaging has the potential to significantly impact treatment planning. The risk of developing metastases is another clinically-relevant factor in determining the best therapy. Niu et al. show that radiomics features from multi-parametric imaging are also potentially able to assess the metastatic risk.

## 4 The broader context

Colorectal cancer is one of the most prevalent cancer types. Local staging, visualization of metastatic spread, and lymph node involvement are crucial not only for prognostic estimation, but also for fully-informed treatment decisions. This is emphasized by the significant understaging currently observed in information obtained from both the primary tumor and nodal involvement, which may, for example, lead to under-identification of patients who would benefit from adjuvant treatment. The articles presented in this Research Topic demonstrate the possible improvements in patient stratification that can be achieved with novel imaging modalities that provide detailed 3D histological images, presenting additional features to routine clinical data that in the future, in combination with deep learning approaches, may be correlated with cancer progression, metastasis, and treatment response. In addition, the increasing use of artificial intelligence has the potential to identify new imaging biomarkers that can provide these predictions even from pre-operative non-invasive imaging technologies.

## 5 Conclusion

This Research Topic is an example of how multiple boundaries are being pushed back, both in terms of imaging technologies and through novel uses of clinical images that are obtained during routine patient care and analyzed retrospectively. To date, the translation of these imaging technologies has been an exciting challenge. For example, the implementation of phase contrast 3D histology into the pathology workflow through reliable small-scale laboratory setups is highly needed. In the case of imaging biomarkers using AI, prognostic prospective validation remains a high bar that only a few studies have cleared. In the future, better subclassification of cancer by novel imaging strategies has the potential to successfully implement personalized therapeutic strategies in order to improve the survival and quality of life of colorectal cancer patients.

## Author contributions

SB: Writing – original draft, Writing – review & editing. FA: Writing – original draft, Writing – review & editing.

